# MXene/MOF-Derived Composites with Multidimensional Nanostructures: Synthesis Methods, Performance, and Applications in the Field of Energy Storage

**DOI:** 10.3390/nano15110841

**Published:** 2025-05-30

**Authors:** Shufan Feng, Shilong Wen, Rutao Wang, Xiaokun Yang, Xiangsen Yuan, Yuxuan Liu, Jingyun Ma, Zhaoqiang Li

**Affiliations:** 1 Shandong Key Laboratory of Chemical Energy Storage and Novel Cell Technology, School of Materials Science and Engineering, Qilu University of Technology (Shandong Academy of Sciences), Jinan 250353, China; fengshufan0810@163.com (S.F.); 10431231187@stu.qlu.edu.cn (Y.L.); 2 Department of Physics, Faculty of Arts and Sciences, Beijing Normal University, Zhuhai 519087, China

**Keywords:** Mxene/MOF-derived, composites, multidimensional nanocomposites, synthesis, properties, applications, energy storage

## Abstract

Metal–organic frameworks (MOFs), formed by the self-assembly of metal ions/clusters and organic linkers, have attracted considerable attention due to their well-exposed active sites, exceptionally high porosity, and diversified pore architectures. MOF-derived materials obtained through high-temperature pyrolysis or composite structural design not only inherit the porous framework advantages of their precursors but also demonstrate significantly enhanced electrical conductivity and structural stability via the formation of carbon-based frameworks and in situ transformation of metallic species. However, conventional MOF-derived materials struggle to address persistent technical challenges in contemporary energy storage systems, particularly those requiring ultralong cycling stability and ultrahigh-rate capability under practical operating conditions. The integration of MXene, characterized by its abundant surface functional groups (-O, -OH, -F) and exceptional electrical conductivity, with MOF-derived materials presents a viable strategy to address these challenges. Multidimensional nanocomposites constructed through in situ growth and self-assembly techniques synergistically integrate MXene’s conductive network scaffolding effect with the structural tunability of MOF-derived frameworks. This unique architecture enables the following: (i) enhanced exposure of electroactive sites, (ii) optimized ion diffusion kinetics, (iii) mechanical integrity maintenance, collectively boosting the applicability of MXene/MOF hybrids in advanced energy storage systems. This review summarizes the synthesis methods, energy storage performance, and applications of multidimensional nanostructured MXene/MOF-derived composites. Finally, it discusses the opportunities and challenges for MXene/MOF-derived composites in future energy storage applications.

## 1. Introduction

MOFs, also termed inorganic–organic hybrids, are emerging crystalline porous materials formed by the coordination coupling of metal ions/clusters with organic ligands. These materials exhibit exceptional advantages, including ultrahigh specific surface area, tunable pore size distributions, and programmable porosity [1,2]. Their derivatives inherit the porous framework advantages of MOF precursors while achieving significantly enhanced electrical conductivity and structural stability through carbon-based matrix formation and in situ transformation of metallic species [3]. Currently, MOF-derived materials have been extensively investigated in energy storage systems such as lithium-ion batteries, supercapacitors, and electrocatalysis [4,5]. However, most conventional MOF derivatives underperform in practical energy storage devices due to insufficient active site accessibility and sluggish ion diffusion kinetics. Incorporating MOFs with functional materials represents an effective strategy to preserve the intrinsic physicochemical characteristics of MOF derivatives while leveraging the complementary advantages of hybrid components and inducing synergistic interfacial effects [6,7].

MXene represent a novel class of two-dimensional (2D) materials composed of surface-modified transition metal carbides, nitrides, and carbonitrides. Their general chemical formula is denoted as M_n+1_X_n_T_x_ (n = 1–4), featuring alternating layers of transition metal (M) atoms, interstitial carbon/nitrogen (X) layers, and surface-terminating functional groups (Tx = -O, -OH, -F) [8,9]. The abundant negatively charged terminal groups enable the formation of interconnected networks through electrostatic interactions, providing rapid ion/electron transport pathways with ionic conductivity exceeding 10³ S cm^−1^ [10,11]. The tunable interlayer spacing accommodates foreign species insertion, facilitating the construction of sandwich-like architectures that mitigate layer restacking issues [12,13]. Furthermore, high-frequency ultrasonication enables the production of few-layer or monolayer MXene nanosheets, which can conformally coat substrates to form core–shell configurations with precisely controlled shell thickness [14,15]. These unique attributes position MXene as ideal candidates for hybridization with MOF-derived materials to achieve synergistic performance enhancement.

This review discusses the synthesis methods, performance in energy storage applications, practical implementations, and future challenges of multidimensional nanostructured MXene/MOF-derived composites. In contrast to existing reviews, this work focuses on the multidimensional nanostructural design of MXene/MOF-derived composites, systematically analyzing their synthesis strategies, interfacial synergistic effects, and optimization mechanisms for enhanced energy storage performance. It is hoped that this review will enhance researchers’ understanding of these materials in the energy storage field.

## 2. Synthesis Method

MOFs, constructed through coordination bonds between metal ions/clusters and organic linkers, serve as ideal precursor materials owing to their structural tunability [16]. MOF-derived nanomaterials have gained significant attention due to their synthetic adaptability and structural diversity, showing exceptional promise for energy storage applications. Synthesized via controlled carbonization of MOF precursors serving as sacrificial templates, these derivatives retain morphological fidelity while acquiring enhanced physicochemical characteristics through pyrolysis [17,18]. Compared to pristine MOFs, the derived materials demonstrate superior electrical conductivity, improved resistance to nanoparticle coalescence, and exceptional structural resilience against electrochemical cycling-induced volume expansion [19].

MXene is a novel 2D layered structural material with advantages such as high conductivity and a large number of active functional groups distributed on its surface [8]. However, the abundant active functional groups on the MXene surface are highly prone to spontaneously react with oxygen-containing groups when exposed to air and aqueous environments, transforming into more stable oxides, resulting in poor oxidation stability of MXene [20]. To enhance the oxidation resistance of MXene, it is necessary to create oxygen-free environments as much as possible during the preparation, processing, and storage of MXene [21]. Preserving MXenes from oxidation is of paramount importance for enhancing their stability and functionality across diverse applications. Multiple strategies have been investigated to inhibit MXene oxidation, including the implementation of protective coatings, antioxidant intercalation, storage in organic solvents, ionic liquid encapsulation, and surface chemical modifications. These approaches not only extend MXenes’ storage longevity but also preserve their intrinsic electronic, mechanical, and chemical properties [22].

The main methods to prevent the oxidation of Mxene are as follows: (i) Storing MXenes in nonpolar or low-polarity organic solvents (e.g., tetrahydrofuran, N-methylpyrrolidone) has emerged as a highly promising solution, as the low concentration of dissolved oxygen in these media significantly retards surface oxidation [23]. (ii) The incorporation of antioxidants serves as an effective strategy for preventing MXene oxidation, where these additives act as protective agents to counteract or inhibit oxidative degradation processes [24]. (iii) Surface encapsulation strategies can be effectively employed to prevent MXene oxidation by ensuring the formation of uniform protective layers on MXene surfaces. These engineered barriers isolate MXenes from oxidative species such as O_2_ and H_2_O [25]. (iv) The stability of MXenes has been significantly enhanced through the utilization of amino acids via hydrogen bonding and coordination interactions, achieving both chemical and colloidal stability. These bonding mechanisms effectively passivate the reactive sites on MXene surfaces, thereby preventing oxidative degradation from water and oxygen molecules [26]. (v) As consistently discussed, the composite formation of MXene with other materials plays a pivotal role in mitigating its inherent oxidation susceptibility. A critical mechanism involves the creation of physical barriers through the incorporation of secondary materials, which restrict MXene’s exposure to external oxidants [27]. Furthermore, certain additives establish robust hydrogen bonding interactions with MXene. These interactions are hypothesized to enhance mechanical strength and flexibility while concurrently suppressing oxidative degradation [28,29].

For the construction of MXene/MOF-derived composite material systems, it is generally necessary to first prepare MXene/MOF composites and then obtain the final MXene/MOF-derived composites through derivation methods. Currently, there are two main preparation strategies: in situ growth strategy (MOFs are grown in situ on the surface and interlayers of MXene to construct MXene/MOF precursor structures, followed by pyrolysis or chemical conversion to derive high-performance MXene/MOF-derived composite systems) and self-assembly strategy (pre-synthesized MOFs and MXene are driven by non-covalent interactions including hydrogen bonding, van der Waals forces, electrostatic interactions, and π-π stacking to spontaneously form stable MXene/MOF precursors, which are then derived using the same methods as the in situ growth strategy to obtain MXene/MOF-derived composites. A minority of preparation strategies involve first converting MOF materials into porous carbon-based materials or metal-based nanocomposites via controlled pyrolysis, followed by composite assembly with MXene nanosheets. Notably, through precise regulation of thermodynamic conversion parameters, MOFs with low intrinsic activity can be effectively transformed into porous carbon-based composites or metal compounds (e.g., metal hydroxides, oxides, sulfides, phosphides, selenides) featuring high specific surface areas and abundant active sites. These strategies enable the formation of integrated multidimensional nanostructures with distinct configurations, fully leveraging synergistic effects between components to significantly enhance the energy storage performance of the composites. Table 1 summarizes the synthesis strategies for MXene/MOF-derived composites with multidimensional nanostructures.

### 2.1. Derived from MXene/MOF Composites

#### 2.1.1. The In Situ Synthesis Method

**Figure 1:**
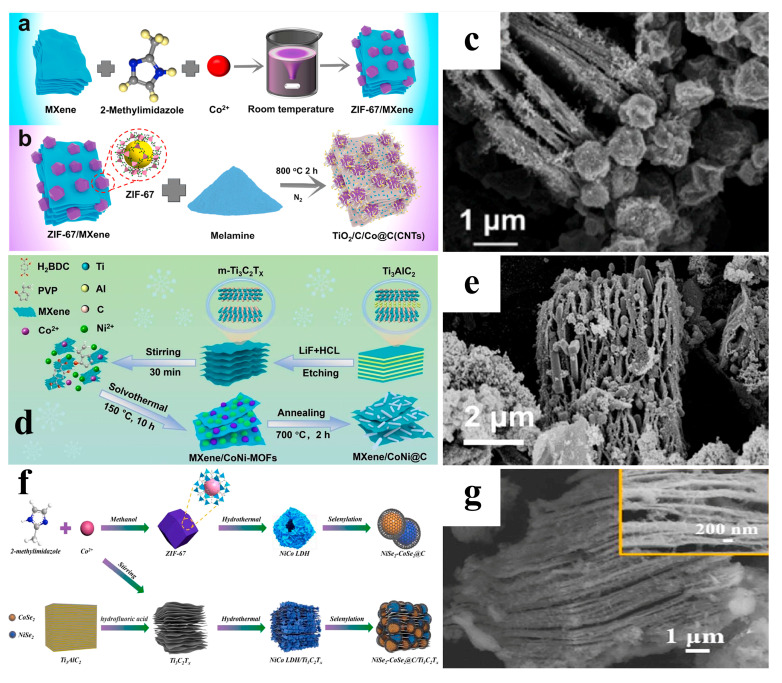
(**a**,**b**) Schematic for preparing of TiO_2_/C/Co@CNTs. (**c**) SEM of TiO_2_/C/Co@CNTs. Adapted from [33], with permission from *Carbon*, 2024. (**d**) Schematic for preparing of MXene/CoNi@C. (**e**) SEM of MXene/CoNi@C. Adapted from [35], with permission from *Journal of Materials Chemistry*, 2024. (**f**) Schematic for preparing of NiSe_2_-CoSe_2_@C/Ti_3_C_2_T_x_. (**g**) SEM of NiSe_2_-CoSe_2_@C/Ti_3_C_2_T_x_. Adapted from [37], with permission from *Chemical Engineering Journal*, 2021.

The in situ synthesis method is one of the most commonly used approaches for preparing MXene/MOF-derived composites. MXene is added to a solution containing fully dissolved metal ions and organic ligands, and the MXene/MOF precursor is prepared using the same experimental methods as those for synthesizing MOFs. Subsequently, the precursor undergoes high-temperature pyrolysis or chemical conversion to derive MXene/MOF-derived composite materials. Due to the spontaneous in situ growth of MOFs on MXene, the derived materials exhibit strong interfacial bonding between the MOF derivatives and MXene, which facilitates synergistic interactions between the two phases to enhance performance [30]. Additionally, the in situ synthesis method simplifies the composite preparation process and reduces resource consumption [31]. Yao et al. employed an in situ growth strategy to synthesize a hybrid aerogel precursor. Specifically, Co(NO_3_)_2_·6H_2_O and 2-methylimidazole were introduced into an aqueous MXene dispersion, forming a hydrogel through coordination-driven self-assembly. Subsequent freeze-drying yielded a ZIF-67@MXene aerogel precursor with a three-dimensional (3D) hierarchical architecture. For sulfur incorporation, the precursor was encapsulated with sulfur powder and subjected to calcination at 600 °C for 2 h under an Ar atmosphere, resulting in the final (CoS NP@HCP)@MXene Aerogel. Structural characterization revealed that the ZIF-67@MXene aerogel comprises highly dispersed ZIF-67 nanoparticles anchored on a 3D MXene framework, establishing a continuous conductive network. Post-thermal treatment, the composite retained a sponge-like morphology with ultralight characteristics, while the ZIF-67-derived CoS nanoparticles (4–7 nm) were uniformly embedded within the nitrogen-doped hierarchically porous carbon (HCP) matrix [32]. Ye’s team prepared ZIF-67/MXene by adding MXene to an aqueous solution containing dissolved Co(NO_3_)_2_·6H_2_O, Ni(NO_3_)_2_·6H_2_O, 2-methylimidazole, followed by continuous stirring at room temperature for 24 h. Subsequently, after thorough mixing with melamine, the material was calcined at 800 °C for 2 h under N_2_ atmosphere to obtain TiO_2_/C/Co@CNTs (Figure 1a,b) [33]. The derived phase from ZIF-67/MXene maintains the accordion-like structure of MXene and the polyhedral structure of ZIF-67, while also containing a large number of in situ-grown carbon nanotubes (CNTs). These components collectively form an integrated multidimensional nano-conductive architecture (Figure 1c). The synthesis processes of MOFs typically require constant temperature and pressure conditions. Therefore, hydrothermal/solvothermal methods have become one of the indispensable approaches for preparing MXene/MOF precursors [34]. Zheng’s team fully dissolved Co(NO_3_)_2_·6H_2_O, Ni(NO_3_)_2_·6H_2_O, PVP and H_2_BDC in a mixed ethanol-DMF solvent at specific ratios, followed by the addition of freeze-dried MXene nanosheets. After hydrothermal treatment to obtain MXene/CoNi-MOF, the material was annealed at 700 °C for 2 h under an Ar atmosphere to yield MXene/CoNi@C (Figure 1d) [35]. The derived CoNi@C is effectively encapsulated and interwoven on the surface and interlayers of MXene, forming a multidimensional complex conductive network (Figure 1e). The room-temperature coprecipitation method offers mild experimental conditions, which helps preserve the structural integrity of materials, simplifies the manufacturing process and reduces costs, while maintaining environmental friendliness [36]. Hou and colleagues synthesized NiSe_2_-CoSe_2_@C/Ti_3_C_2_T_x_ through the following protocol: Co(NO_3_)_2_·6H_2_O was introduced into a methanol solution containing ultrasonically dispersed 2-methylimidazole and MXene, followed by room-temperature coprecipitation to form Co-MOF/Ti_3_C_2_T_x_ precursors. The precursors were redispersed with Ni(NO_3_)_2_·6H_2_O in absolute ethanol for hydrothermal treatment at 120 °C for 8 h, then collected via centrifugation and dried overnight to obtain NiCo LDH/Ti_3_C_2_T_x_. Final selenization yielded NiSe_2_-CoSe_2_@C/Ti_3_C_2_T_x_ (Figure 1f) [37]. The NiCo LDH exhibited amorphous flocculent deposits on the accordion-like MXene layers without defined morphology. Post-selenization, these deposits transformed into discrete nanoparticles homogeneously distributed across the MXene scaffold (Figure 1g).

#### 2.1.2. The Self-Assembly Method

**Figure 2:**
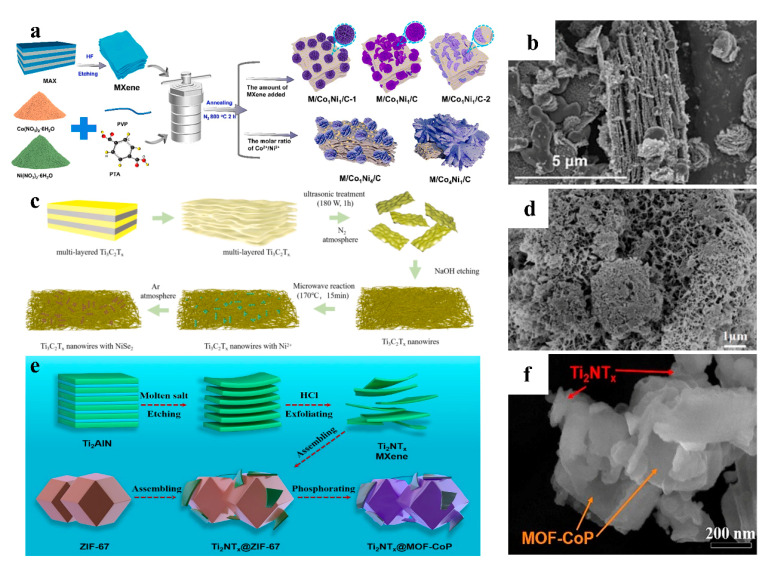
(**a**) Schematic for preparing of MXene/CoNi/C. (**b**) SEM of MXene/CoNi/C. Adapted from [40], with permission from *Chemical Engineering Journal*, 2023. (**c**) Schematic for preparing of NiSe_2_/NC/Ti_3_C_2_T_x_. (**d**) SEM of NiSe_2_/NC/Ti_3_C_2_T_x_. Adapted from [41], with permission from *Applied Surface Science*, 2025. (**e**) Schematic for preparing of Ti_2_NT_x_@MOF-CoP. (**f**) TEM of Ti_2_NT_x_@MOF-CoP. Adapted from [42], with permission from *Electrochimica Acta*, 2021.

The self-assembly method can be driven by forces such as electrostatic interactions, π-π stacking, and hydrogen bonding, enabling the spontaneous formation of ordered structures. Compared with traditional preparation processes, this method for synthesizing MXene/MOF derivatives overcomes the constraints of precursor material synthesis conditions. While preserving the intrinsic properties of the materials, it demonstrates significant process controllability and application adaptability [38,39]. Lv and his team first prepared gray CoNi-MOF powder in specific proportions. Subsequently, MXene was added in varying amounts for self-assembly with the MOF precursor, yielding MXene/CoNi-MOF. Finally, the MXene/CoNi/C composite series was obtained through high-temperature calcination under an inert atmosphere (Figure 2a) [40]. With increasing molar Co^2+^ and Ni^2+^, the CoNi-MOF transformed from nanosheet-like structures into petal-like morphologies, accompanied by increased petal thickness and overall dimensions. After high-temperature annealing, the original accordion-like morphology of MXene remained intact, while the CoNi-MOF derivatives evolved into CoNi alloys and graphitic carbon, exhibiting progressively roughened surfaces (Figure 2b). Zhou et al. synthesized Ni MOF-Ti_3_C_2_T_x_ composites via a microwave-assisted self-assembly technique and further derived them into NiSe_2_/NC/Ti_3_C_2_T_x_ for electromagnetic wave absorption. The synthetic procedure is outlined as follows. First, a specific molar ratio of Co(NO_3_)_2_·6H_2_O, maleic hydrazide, and PVP was dissolved in a mixed solvent of water and DMF. Subsequently, varying amounts of alkali-treated MXene nanowires were introduced into the solution. The mixture was subjected to microwave irradiation at 170 °C with a power of 150 W for 15 min to yield Ni MOF-Ti_3_C_2_T_x_ composites. Finally, the obtained composites were selenized at 600 °C for 2 h under an inert atmosphere to produce NiSe_2_/NC/Ti_3_C_2_T_x_ (Figure 2c) [41]. Through microwave-assisted self-assembly, petal-like thin films of Ni-MOF were successfully anchored onto MXene nanowires. After high-temperature selenization, the original flower-like architecture collapsed, resulting in uniformly distributed and densely packed nanoparticles on both the surface and interlayers of MXene. This structural evolution formed an intricate 0D-3D porous network with enhanced complexity (Figure 2d). Zong’s team employed a self-assembly strategy to synthesize Ti_2_NT_x_@MOF-CoP by introducing Ti_2_NT_x_ nanosheets into a ZIF-67-dispersed suspension under continuous stirring, yielding a Ti_2_NT_x_@ZIF-67 precursor. Prior to phosphidation, the precursor underwent decarbonization in a tube furnace at 450 °C for 1 h. Subsequently, an appropriate amount of NaH_2_PO_2_·H_2_O was placed upstream in an argon flow, and the intermediate was phosphidized at 350 °C to obtain Ti_2_NT_x_@MOF-CoP (Figure 2e) [42]. The phosphidized MOF-CoP exhibited a well-defined cubic microstructure, with ultrathin layered Ti_2_NT_x_ nanosheets uniformly enveloping the MOF-CoP surfaces. Additionally, Ti_2_NT_x_ nanosheets were interspersed between MOF-CoP particles, enhancing interfacial bonding strength among adjacent nanoparticles (Figure 2f).

### 2.2. Integration of MOF-Derived Materials with MXene

Compared to directly deriving MXene/MOF composites from MXene/MOF precursors, the strategy of first derivatizing MOFs into their derivatives followed by hybridization with MXene offers two critical advantages: (i) it circumvents potential structural degradation of MXene during harsh derivation processes, and (ii) it enables precise control over the structural and phase composition of MOF-derived materials. This sequential approach facilitates application-oriented material design, thereby allowing targeted enhancement of desired properties through rational architectural engineering [43].

**Figure 3:**
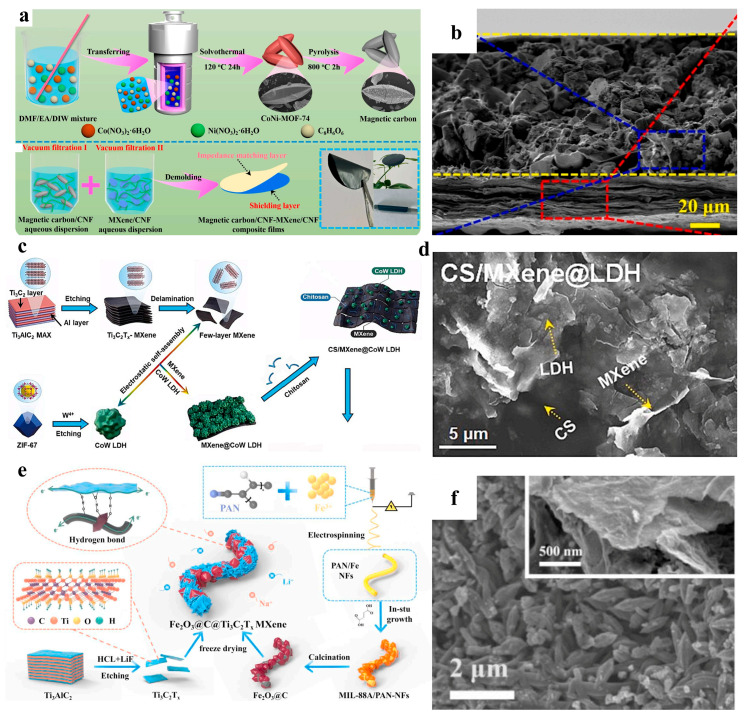
(**a**) Schematic for preparing of magnetic carbon/CNF-MXene/CNF composite films. (**b**) SEM of magnetic carbon/CNF-MXene/CNF composite films. Adapted from [44], with permission from *Chemical Engineering Journal*, 2024. (**c**) Schematic for preparing of CS/MXene@CoW LDH. (**d**) SEM of CS/MXene@CoW LDH. Adapted from [45], with permission from *Chemical Engineering Journal*, 2024. (**e**) Schematic for preparing of Fe_2_O_3_@C@Ti_3_C_2_T_x_ MXene. (**f**) SEM of Fe_2_O_3_@C@Ti_3_C_2_T_x_ MXene. Adapted from [46], with permission from *Journal of Power Sources*, 2024.

Guo et al. synthesized CoNi-MOF-74 precursors via a solvothermal method and subsequently calcined them to obtain spindle-shaped derived magnetic carbon. The magnetic carbon was ultrasonically treated with cellulose nanofibrils (CNFs) to form stable magnetic carbon/CNF hydrogels. MXene nanosheets were uniformly mixed with CNFs via vacuum filtration and deposited atop the magnetic carbon/CNF substrate, yielding the final magnetic carbon/CNF-MXene/CNF composite films [44]. This uniquely designed multidimensional nanostructured composite film significantly improves impedance matching due to the rational arrangement of components, effectively promoting the entry and dissipation of incident electromagnetic waves. The constructed bilayer architecture induces an “absorption-reflection-reabsorption” mechanism, markedly enhancing electromagnetic interference (EMI) shielding effectiveness. Furthermore, abundant hydrogen bonding interactions and tight interfacial bonding endow the composite films with exceptional mechanical flexibility, satisfying practical application demands and broadening their application prospects in wearable electronics and aerospace shielding systems. Kasinathan’s team synthesized chitosan-functionalized CS/MXene@CoW LDH by first adding a controlled amount of Na_2_WO_4_·2H_2_O to a ZIF-67-dispersed anhydrous ethanol solution under continuous stirring, utilizing ZIF-67 as a sacrificial template to form CoW layered double hydroxide with a hollow polyhedral structure. The resulting CoW LDH was then introduced into an aqueous suspension containing few-layer MXene nanosheets, subjected to 1 h ultrasonication, and centrifuged to obtain MXene@CoW LDH intermediates. Finally, the intermediates were thoroughly mixed with chitosan (CS) and hydrothermally processed to yield CS/MXene@CoW LDH (Figure 3c) [45]. The CoW LDH hollow polyhedra, successfully templated by ZIF-67, retained the identical morphology and dimensions as the precursor ZIF-67. Both few-layer MXene nanosheets and LDH were homogeneously distributed within the chitosan matrix, creating an integrated hierarchical architecture (Figure 3d). Li et al. first fabricated MIL-88A/polyacrylonitrile nanofibers (MIL-88A/PAN-NFs) via electrospinning, which were subsequently calcined to derive Fe_2_O_3_@C. The Fe_2_O_3_@C was then co-dispersed with MXene nanosheets in deionized water under ultrasonication, followed by freeze-drying to obtain bead-like Fe_2_O_3_@C@Ti_3_C_2_T_x_ MXene hybrids (Figure 3e) [46]. The Ti_3_C_2_T_x_ MXene flakes uniformly encapsulated the Fe_2_O_3_@C nanofibers, with the nanofibers longitudinally penetrating the bead-like structures. Furthermore, the inherently porous architecture of the beads facilitated electrolyte infiltration and enhanced ion migration kinetics (Figure 3f). The XPS analysis of the FCM-6 composite reveals that its hierarchically designed nanostructure synergistically enhances electrochemical performance through multiscale electronic and ionic transport optimization. The deconvoluted C 1s spectrum demonstrates critical interfacial bonding configurations, where the C-Ti bond at 282.4 eV confirms strong covalent coupling between carbon nanofibers and Ti_3_C_2_T_x_ MXene nanosheets, establishing continuous conductive pathways essential for rapid charge transfer. Nitrogen-doping-induced defects, evidenced by pyridinic-N (396.4 eV) and pyrrolic-N (398.1 eV) configurations, create electrochemically active sites while improving electronic conductivity through π-electron system modulation. These structural modifications in the 1D carbon nanofiber matrix facilitate Li^+^/Na^+^ intercalation kinetics by reducing diffusion barriers at the nanoscale. The interfacial engineering is further evidenced in the O 1s spectrum, where Fe-O (529.1 eV) and Ti-O (534.2 eV) bonds indicate heterointerface formation between Fe_2_O_3_ nanoparticles and MXene nanosheets. This nanoscale hybridization leverages the pseudocapacitive properties of MXene and conversion-type Fe_2_O_3_ through optimized electron transfer at phase boundaries. The Ti 2p spectrum confirms preserved MXene characteristics with Ti-C bonds (454.4/460.8 eV), maintaining its 2D layered conductivity while surface oxygenated groups (-F, -OH) enable hydrogen bonding with Fe_2_O_3_ during freeze-drying. This 0D-2D-1D nanoarchitecture creates a porous conductive network with shortened ion diffusion lengths and enhanced structural resilience. Notably, the nanoconfined Fe_2_O_3_ particles mitigate pulverization stresses during cycling, while MXene nanosheets act as buffering matrices. The synergistic defect engineering and interfacial charge redistribution collectively improve rate capability and cycling stability, demonstrating how multiscale nanostructuring optimizes both thermodynamic and kinetic properties in hybrid electrode systems.

**Table 1:** Summary of the synthesis strategies of MXene/MOF-derived composites.

Material	Preparation	Assisting Method	Solvent	Ref.
TiO_2_/C/Co@CNTs	In situ synthesis	Hydrothermal Pyrolysis (800 °C, N_2_, 2 h)	DI	[33]
MXene/CoNi@C	In situ synthesis	Solvothermal Pyrolysis (700° C, Ar, 2 h)	Ethanol, DMF	[35]
NiSe_2_-CoSe_2_@C/Ti_3_C_2_T_x_	In situ synthesis	Solvothermal Selenylation (400° C, Ar, 2 h)	MeOH	[37]
M/CoNi/C	Self-assembly	Solvothermal Pyrolysis (800° C, N_2_, 2 h)	Ethanol, DMF	[40]
NiSe_2_/NC/Ti_3_C_2_T_x_	Self-assembly	Microwave-facilitated Solvothermal (600° C, Ar, 2 h)	DI, DMF	[41]
Ti_2_NT_x_@MOF-CoP	Self-assembly	Stirring Phosphating (300° C, Ar, 1.5 h)	DI	[42]
Carbon/CNF-MXene/CNF composite films	Direct mixing	Solvothermal Pyrolysis (800° C, N_2_, 2 h) vacuum-assisted filtration	DI, DMF, Ethanol	[44]
MXene@LDH hybrid NCs	Self-assembly	Stirring Solvothermal	DI, Ethanol	[45]
MOF-Fe_2_O_3_@carbon@Ti_3_C_2_T_x_ MXene	Direct mixing	Electrospinning Hydrothermal Pyrolysis (600° C, N_2_, 2 h)	DMF, DI	[46]

## 3. Performance

Pure MOF derivatives are plagued by inherent limitations such as poor structural stability, low electrical conductivity, and inadequate rate capability, which severely restrict their large-scale applications in energy storage systems. MXene, with its exceptional electrical conductivity and abundant surface functional groups, serves as an ideal functional counterpart for hybridization with MOF derivatives. This multidimensional nanocomposite architecture not only effectively mitigates the intrinsic drawbacks of MOF derivatives in energy storage applications but also generates synergistic effects through interfacial interactions, thus significantly enhancing overall performance metrics including charge transfer kinetics, structural integrity, and electrochemical cyclability.

### 3.1. Stabilities

**Figure 4:**
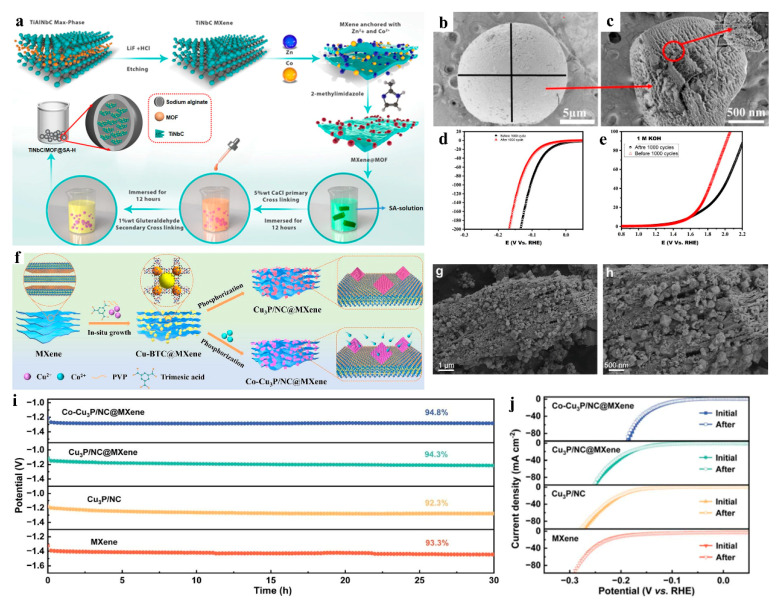
(**a**) Schematic for preparing of MXene@MOF/SA@H hydrogel 3D framework. (**b**,**c**) SEM of MXene@MOF/SA@H hydrogel 3D framework. (**d**,**e**) HER and OER polarization LSV curve before and after 1000 CV cycles. Adapted from [47], with permission from *Journal of Materials Chemistry A*, 2025. (**f**) Schematic for preparing of 3D@2D Co-Cu_3_P/NC@MXene. (**g**,**h**) SEM of 3D@2D Co-Cu_3_P/NC@MXene. (**i**) CP curves and (**j**) initial and final LSV curves for 1000 CV cycles. Adapted from [48], with permission from *Journal of Materials Chemistry A*, 2024.

Certain MOF derivatives frequently suffer from poor stability during practical applications. Through rational engineering of multidimensional nanostructures and precise phase composition in MXene/MOF derivative composites, synergistic interfacial interactions can be achieved, thereby substantially enhancing structural stability while maintaining electrochemical activity under operational stresses. Raza et al. reported a multifunctional MXene@MOF/SA@H hydrogel hierarchical 3D framework—a novel integrated multidimensional nanostructured material—as a bifunctional catalyst for hydrogen evolution reaction (HER) and oxygen evolution reaction (OER), demonstrating its potential as a high-performance electrocatalyst for energy conversion applications (Figure 4a–c). The material exhibited exceptional catalytic activity with Tafel slopes of 61.8 mV dec-1 for HER and 84 mV dec-1 for OER. Notably, it retained performance integrity over 1000 cyclic stability tests within 24 h while maintaining negligible mass loss, underscoring its remarkable operational durability (Figure 4d,e) [47]. Zhang’s team developed a novel 3D@2D hierarchical Co-Cu_3_P/NC@MXene hybrid nanocatalyst by in situ growth of octahedral co-doped CU-BTC on MXene followed by high-temperature phosphidation (Figure 4f–h). This unique 3D@2D architecture not only prevents self-agglomeration of Co-Cu_3_P/NC nanoparticles while preserving their well-dispersed configuration but also enhances catalytic activity and electrochemical stability during the HER, as evidenced by its sustained performance under prolonged operational conditions (Figure 4i,j) [48].

**Figure 5:**
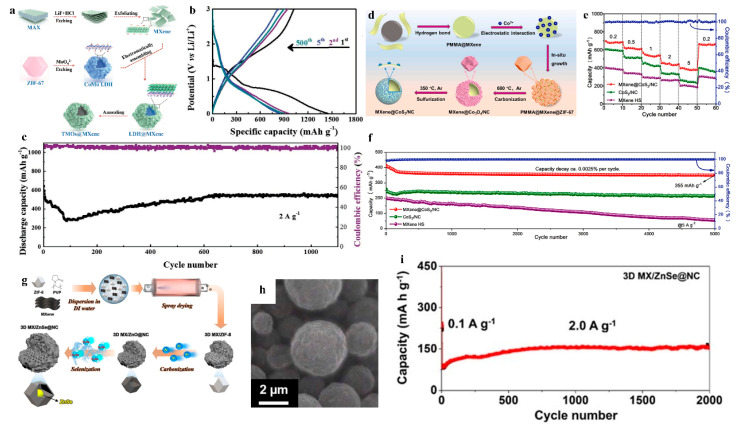
(**a**) Schematic for preparing of CoO/Co_2_Mo_3_O_8_@MXene. (**b**) GCD profiles at the 1st, 2nd, 5th, and 500th cycles under 0.1 mV s^−1^ of hollow CoO/Co_2_Mo_3_O_8_@MXene polyhedrons. (**c**) Cycling behaviors of CoO/Co_2_Mo_3_O_8_ @MXene electrode over 1200 cycles at 2 A g^−1^. Adapted from [49], with permission from *Small*, 2019. (**d**) Schematic for preparing of MXene@CoS_2_/NC. (**e**) Rate capability of MXene@CoS_2_/NC at different current densities. (**f**) Long-term cycling stability of MXene@CoS_2_/NC. Adapted from [50], with permission from *Chemical Engineering Journal*, 2022. (**g**) Schematic for preparing of 3D MX/ZnSe@NC. (**h**) SEM of 3D MX/ZnSe@NC and (**i**) long-term cycling performance at 2.0 A g^−1^. Adapted from [51], with permission from *Journal of Materials Chemistry A*, 2024.

The multidimensional nanostructure of MXene/MOF derivative composites confers exceptional structural stability, effectively mitigating deformation caused by repeated insertion/extraction of metal ions (e.g., Li^+^, Na^+^, K^+^) during cycling. This architectural robustness substantially enhances cycling stability by preserving structural integrity against lattice strain and volume fluctuations inherent to ion transport processes. Zhao and colleagues employed an electrostatic self-assembly strategy to integrate ZIF-67-derived CoMo layered double hydroxide with MXene nanosheets, followed by annealing to synthesize CoO/Co_2_Mo_3_O_8_@MXene composites (Figure 5a). Within this uniquely designed hollow hierarchical architecture, MXene nanosheets form strong interfacial bonds with the LDH derived from ZIF-67, establishing a stable three-dimensional conductive network. This structural configuration effectively mitigates structural deformation and damage induced by repeated Li^+^ insertion/extraction during charge/discharge cycles, thereby significantly enhancing cycling stability (Figure 5b,c) [49]. Li et al. fabricated MXene@CoS_2_/NC composites for sodium-ion battery anodes by first employing polymethyl methacrylate (PMMA) as a sacrificial template to assemble MXene hollow spheres (HS). Subsequent in situ growth of ZIF-67 followed by high-temperature carbonization and sulfidation yielded CoS_2_/NC heterostructures anchored on the HS (Figure 5d). The unique hollow nanostructure and engineered heterojunction interfaces weakened Co-S bond energy, which synergistically enhanced sodium-ion storage capabilities. This structural optimization resulted in ultrahigh specific capacity (exceeding 704 mAh g-1 at 0.2 A g-1) and exceptional cycling stability (92% capacity retention after 5000 cycles), outperforming conventional CoS_2_-based anodes (Figure 5e,f) [50]. Jeong’s team synthesized ZnSe@NC nanoparticles encapsulated within 3D MXene microspheres (3D MX/ZnSe@NC) as an advanced potassium-ion battery anode through a multi-step protocol. First, pre-synthesized MXene nanosheets, ZIF-8 particles, and polyvinylpyrrolidone (PVP) were homogeneously dispersed in deionized water under stirring, followed by spray-drying to fabricate 3D MXene/ZIF-8 precursors. Subsequent high-temperature calcination and selenization yielded the final 3D MX/ZnSe@NC architecture (Figure 5g,h). The synergistic integration of MXene and N-doped carbon (NC) in this hybrid establishes a continuous conductive network that accelerates electron/ion transport kinetics. The three-dimensional microspherical framework constructed from 2D MXene nanosheets effectively inhibited MXene restacking while the MXene encapsulation effectively mitigated volume variation of ZnSe nanocrystals during prolonged cycling, preventing structural collapse. This hierarchical design endowed the material with exceptional cyclability, maintaining 88% capacity retention after 1000 cycles at 2 A g-1 (Figure 5i) [51].

### 3.2. Ion/Electron Transport Rate

MOF derivatives, typically comprising porous carbon-based composites or metal compounds, are fundamentally constrained by their limited electrical conductivity and insufficient density of active sites, failing to meet the escalating performance demands in energy storage applications. In contrast, MXene/MOF derivative composites with multidimensional nanostructures exhibit interconnected conductive networks that significantly enhance bulk conductivity through synergistic electron transport pathways [52]. Furthermore, the strategic intercalation of MOF-derived components into MXene interlayers can effectively mitigate the self-restacking of MXene while exposing additional electrochemically active sites. This structural engineering strategy simultaneously improves electrolyte permeability and establishes continuous ion/electron transport pathways, thereby accelerating charge transfer kinetics by optimizing interfacial ion diffusion and electron hopping mechanisms.

**Figure 6:**
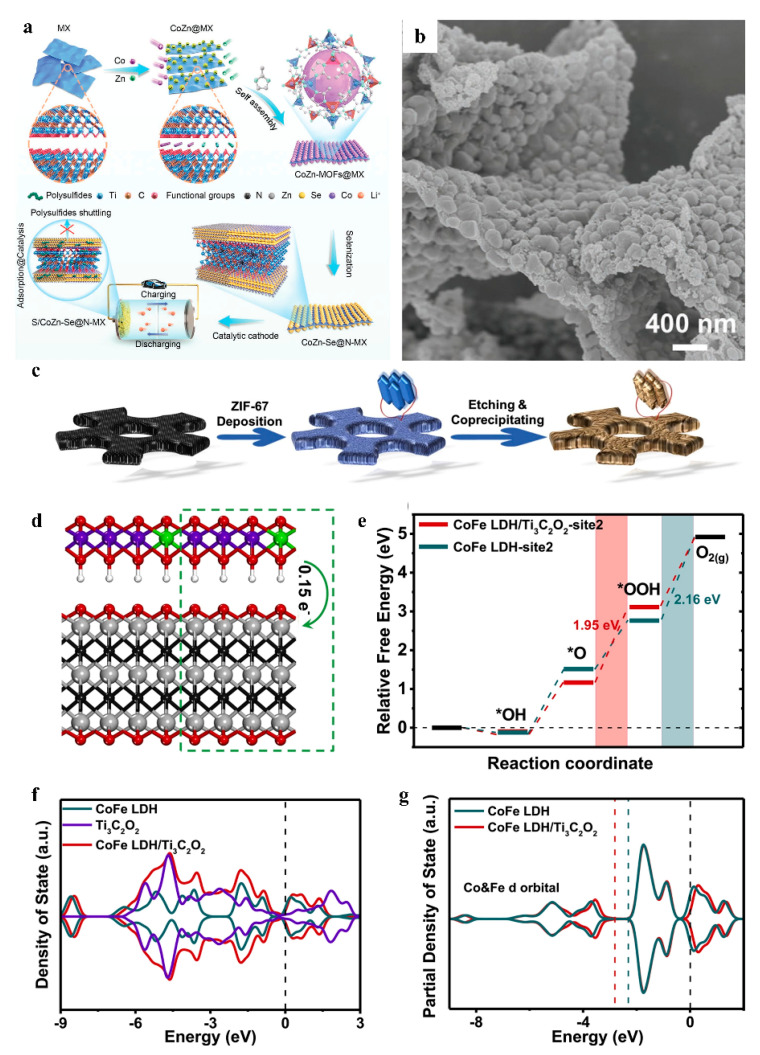
(**a**) Schematic for preparing of CoZnSe@N-MX. (**b**)SEM of CoZnSe@N-MX. Adapted from [53], with permission from *Advanced Materials*, 2021. (**c**) Schematic for preparing of CoFe MLDH/Mxene/NF. (**d**) Side view of the optimized CoFe LDH/Ti_3_C_2_O_2_ with 2 × 1 × 1 crystal cell (H: white, C: black, O: red, Ti: silver, Co: purple and Fe: green). (**e**) Free energy diagrams at 0 V for the OER processes on the surface of individual CoFe LDH and CoFe LDH/Ti3C_2_O_2_ composite. (**f**) DOS of CoFe LDH/Ti3C_2_O_2_ composite, individual CoFe LDH and Ti_3_C_2_O_2_ with Fermi level at zero (black dashed line). (**g**) PDOS (3d orbitals of Co and Fe) of bare CoFe LDH and CoFe LDH/Ti_3_C_2_O_2_ (blue and red dashed lines: d-band center). Adapted from [54], with permission from *Applied Catalysis B: Environmental*, 2021.

Ye et al. fabricated bimetallic selenide@nitrogen-doped MXene (CoZnSe@N-MX) with multicomponent heterostructures via a self-assembly strategy. The CoZn-Se nanoparticles were uniformly distributed on the surface and interlayers of N-MX nanosheets, effectively preventing their restacking and thereby creating abundant electrochemically active channels for enhanced ion/electron transport. Furthermore, the abundant functional groups on the N-MX surface strengthened the interfacial coupling between CoZn-Se nanoparticles and N-MX, significantly shortening ion/electron diffusion pathways (Figure 6a,b). This synergistic structural design resulted in accelerated charge transfer kinetics, as evidenced by a 2.3-fold improvement in ionic conductivity compared to pristine MXene-based counterparts [53]. Furthermore, the enhancement of ion/electron transport kinetics in MXene/MOF-derived composite nanomaterials can be mechanistically validated through density functional theory (DFT) simulations. Hu and colleagues fabricated layered CoFe MLDH/Ti_3_C_2_/NF, derived from ZIF-67/Ti_3_C_2_/NF via an in situ growth strategy, as an oxygen evolution reaction (OER) catalyst (Figure 6c). DFT calculations revealed that the exceptional OER performance originates from weakened adsorption strength of reaction intermediates on CoFe MLDH/Mxene/NF compared to pristine CoFe LDH, resulting in a reduced reaction energy barrier. Moreover, MXene induces electronic structure modulation by facilitating electron redistribution from the CoFe LDH layers to MXene, as evidenced by charge density difference plots and Bader charge analysis. The precisely engineered nanoarchitecture of the CoFe LDH/Ti_3_C_2_O_2_ heterostructure fundamentally enhances electrocatalytic performance through atomic-level structural modulation and interfacial synergy. The orthorhombic lattice configuration (*a* = 5.26 Å, *b* = 6.08 Å) with well-defined 1.64 Å interlayer spacing (Figure 6d) establishes nanoscale charge transport channels that facilitate rapid ion diffusion while maintaining structural integrity, as evidenced by the substantial binding energy. The interfacial electron redistribution, revealed by Hirshfeld charge analysis (0.15 *e* depletion from CoFe LDH to Ti_3_C_2_O_2_), creates built-in electric fields at the 2D–2D heterointerface that significantly accelerate charge transfer kinetics, a critical advantage enabled by nanoscale layer engineering. The OER performance enhancement originates from precisely tailored adsorption energetics at atomic active sites. The reduced energy barrier for *OOH intermediate formation (1.95 eV vs. 2.16 eV in pristine LDH) stems from optimized d-band center positioning, where the composite’s Co/Fe d-band centers shift downward by 0.5 eV compared to standalone LDH. This nanoscale electronic structure modification, corroborated by DOS/PDOS analysis (Figure 6f,g), weakens intermediate adsorption through strategic orbital hybridization while maintaining sufficient activation capability. The heterostructure’s expanded surface states near the Fermi level, arising from Ti_3_C_2_O_2_-induced electron delocalization, provide abundant catalytic sites with optimized charge distribution. Notably, the nanoconfined interface prolongs intermediate residence time through spatially controlled adsorption/desorption dynamics, enabling efficient four-electron pathway completion. This atomic-level synergy between LDH’s redox-active sites and MXene’s conductive nanosheets exemplifies how precisely engineered 2D hetero-nanoarchitectures can surpass single-component systems by simultaneously optimizing electronic structure, mass transport, and reaction thermodynamics [54].

### 3.3. Pseudocapacitance

**Figure 7:**
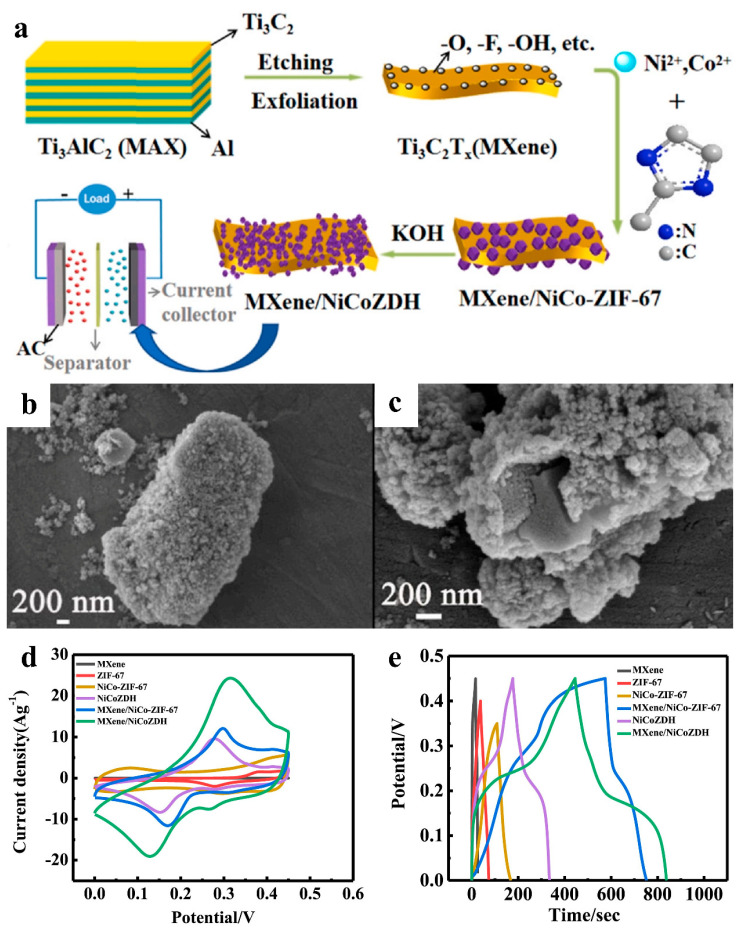
(**a**) Schematic for preparing of MXene/NiCoZDH. (**b**,**c**) SEM of MXene/NiCoZDH. (**d**) CV curves of MXene, ZIF-67, NiCo-ZIF-67, NiCoZDH, MXene/NiCo-ZIF-67 and MXene/NiCoZDH recorded at a scan rate of 20 mV s^−1^. (**e**) GCD diagrams of MXene, ZIF-67, NiCo-ZIF-67, NiCoZDH, MXene/NiCo-ZIF-67 and MXene/NiCoZDH at 1 A g^−1^. Adapted from [55], with permission from *Journal of Alloys and Compounds*, 2021.

MXene’s lamellar structure and high electrical conductivity facilitate rapid electrolyte penetration through its stacked layers, while its abundant surface-bound active functional groups enable high-rate reversible redox reactions. When hybridized with MOF derivatives, this composite architecture synergistically introduces pseudocapacitive contributions through interfacial charge redistribution and enhanced Faradaic activity at the MXene/MOF derivative heterointerfaces. Guo et al. developed MXene/NiCoZDH composite nanomaterials for high-performance, long-cycle-life supercapacitors by alkaline treatment of in situ-grown MXene/NiCo-ZIF-67 precursors (Figure 7a–c). The NiCoZDH was chemically anchored onto MXene surfaces via robust covalent bonding, which not only prevented active material detachment but also exposed abundant electrochemically active sites. Electrochemical characterization revealed that the MXene/NiCoZDH hybrid exhibited the highest specific capacitance among all control samples, demonstrating MXene’s critical role in enhancing capacitive performance through synergistic interfacial charge storage and improved ion accessibility (Figure 7d,e) [55].

### 3.4. Rate Performance

The exploration of high-rate-performance materials is critically important for advancing energy storage systems toward practical applicability [56]. The multidimensional nanostructured MXene/MOF-derived composites exhibit multifunctional synergistic effects in conductive network construction, ion transport optimization, and structural compatibility.

Leveraging its high electrical conductivity and two-dimensional layered architecture, MXene establishes a continuous conductive framework that shortens electron transport pathways, while its surface hydrophilic functional groups enhance electrolyte wettability. The MOF-derived components, obtained through high-temperature carbonization, possess porous nanostructures that synergize with MXene’s interlayer mesopores to form hierarchical pore channels. This architecture significantly reduces ion/electron diffusion distances and lowers migration energy barriers. Furthermore, MXene’s inherent flexibility and mechanical robustness effectively buffer the volume expansion of MOF derivatives during charge/discharge cycles, thereby preserving electrode structural integrity. Chemical bonding at the MXene/MOF-derived heterointerfaces further optimizes the electronic structure of active sites by inducing charge redistribution, which lowers the activation energy of electrochemical reactions. These multiscale synergistic mechanisms enable MXene/MOF-derived composites to maintain rapid charge transfer kinetics even under high current densities, effectively addressing the intrinsic limitations of pure MOF derivatives (poor conductivity) and pure MXene (structural instability). Consequently, the composites demonstrate exceptional rate capability.

**Figure 8:**
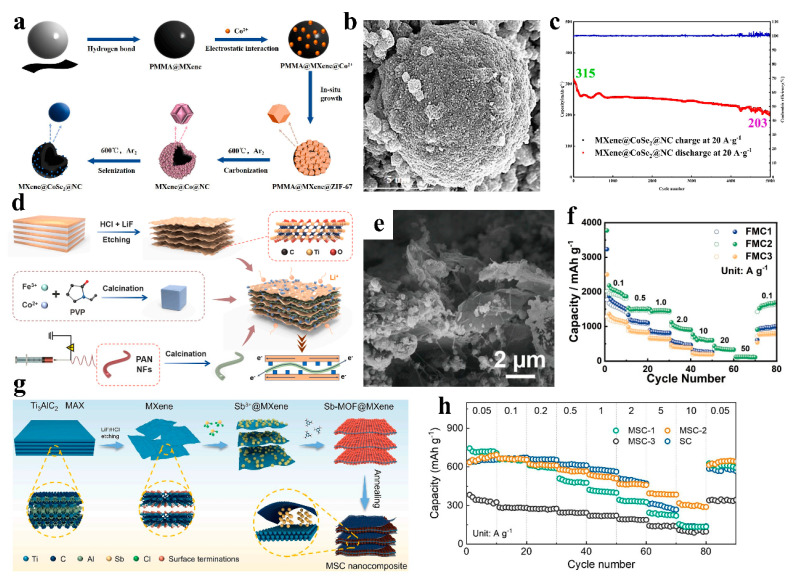
(**a**) Schematic for preparing of MXene@CoSe_2_@NC. (**b**) SEM of MXene@CoSe_2_@NC. (**c**) Enhanced durability of MXene@CoSe_2_@NC during cycling at a high current density of 20 A g^−1^. Adapted from [57], with permission from *Journal of Energy Storage*, 2024. (**d**) Schematic for preparing of FMC. (**e**) SEM of FMC. (**f**) Rate performance of FMC. Adapted from [58], with permission from *Journal of Alloys and Compounds*, 2024. (**g**) Schematic for preparing of MSC. (**h**) Rate performance of MSC. Adapted from [59], with permission from *Chemical Engineering Journal*, 2024.

An et al. synthesized multidimensional hollow microspheres (MXene@CoSe_2_@NC) through a sequential protocol. First, PMMA microspheres surface-coated with MXene nanosheets (PMMA@MXene) were fabricated via a self-assembly strategy. ZIF-67 was then grown in situ on these microspheres to form PMMA@MXene@ZIF precursors. Subsequent removal of PMMA via thermal decomposition, followed by carbonization and selenization, yielded the final MXene@CoSe_2_@NC 0D-3D architecture with hierarchical porosity (Figure 8a,b). As a sodium-ion battery anode, MXene@CoSe_2_@NC demonstrated remarkable cycling stability, retaining a capacity of 203 mAh g^−1^ after 5000 cycles at an ultrahigh current density of 20 A g^−1^, with a moderate decay from the initial 315 mAh g^−1^—equivalent to an average capacity loss of only 0.71% per 100 cycles (Figure 8c). This exceptional rate performance originates from the robust interfacial coupling between surface-active CoSe_2_@NC nanoparticles and the conductive MXene hollow sphere (HS) substrate. The three-dimensional configuration effectively mitigates volume expansion and mechanical stress during sodiation/desodiation, while the interconnected MXene framework ensures rapid Na^+^ diffusion and electron transfer [57]. Dai and colleagues synthesized a hierarchically structured composite (denoted as FMC) through a multi-stage assembly strategy. First, MOF-derived CoFe_2_O_4_ nanoparticles (MOF-CoFe_2_O_4_) were integrated with Ti_3_C_2_T_x_ MXene nanosheets via self-assembly to form an intermediate MOF-CoFe_2_O_4_/Ti_3_C_2_T_x_ MXene. Subsequently, carbon nanofibers (CNFs), derived from electrospun polymer fibers through high-temperature carbonization, were ultrasonically hybridized with the intermediate to yield the final FMC architecture (Figure 8d,e). The MOF-derived CoFe_2_O_4_ nanoparticles not only delivered high theoretical capacity but also occupied MXene nanosheet surfaces and interlayers, effectively preventing MXene restacking while preserving its structural and mechanical integrity. MXene’s incorporation substantially enhanced bulk conductivity, reducing charge transfer resistance and shortening ion/electron diffusion pathways. The introduced CNFs further established an interpenetrated conductive network, exposing additional active sites and buffering volumetric strain during lithiation/delithiation. When evaluated as a lithium-ion battery anode, the FMC composite exhibited exceptional rate capability, retaining a capacity of 332 mAh g^−1^ at an ultrahigh current density of 20 A g^−1^ (Figure 8f) [58]. Feng’s team synthesized MXene-reinforced Sb@C nanocomposites (MSC) by first growing Sb-MOF in situ on MXene nanosheets (Sb-MOF@MXene) via a coordination-assisted strategy, followed by calcination under an inert atmosphere (Figure 8g). The composite features a synergistic conductive network comprising MXene’s ultrahigh-conductivity substrate and MOF-derived carbon layers. Owing to its spatially confined architecture, the MSC framework not only enhances electrical conductivity but also accommodates volume variations of Sb nanoparticles during lithiation/delithiation, thereby preserving structural integrity and electrochemical activity. This nanoscale engineering yielded exceptional rate capability, with the MSC anode retaining a specific capacity of 305 mAh g^−1^ at 10 A g^−1^ (Figure 8h) [59].

## 4. Applications

### 4.1. Electrocatalysts

Research on electrocatalysts for the hydrogen evolution reaction (HER), oxygen evolution reaction (OER), and oxygen reduction reaction (ORR) is pivotal to advancing energy storage and conversion systems [60,61,62]. MXene, with its high electrical conductivity, abundant surface functional groups, and tunable interlayer spacing, acts as an effective co-catalyst by modulating the electrophilicity of active sites to enhance catalytic efficiency [63]. Furthermore, the multidimensional nanostructures of MXene/MOF-derived composites synergistically combine high stability with rapid ion/electron transport kinetics, achieved through hierarchical porosity and interfacial charge redistribution. These attributes enable precise control over reaction pathways and intermediate adsorption energetics, making them ideal electrocatalysts for next-generation energy technologies.

**Figure 9:**
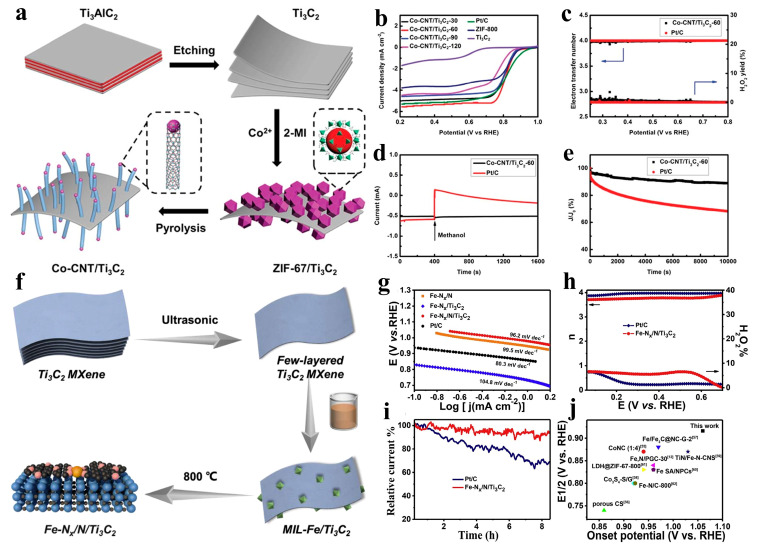
(**a**) Schematic for preparing of Co-CNT/Ti_3_C_2_. (**b**) LSV curves of Co-CNT/Ti_3_C_2_, Pt/C, ZIF-800 and Ti_3_C_2_. (**c**) The yield of H_2_O_2_ and electron transfer numbers of Co-CNT/Ti_3_C_2_-60 and Pt/C. (**d**) Methanol crossover resistance of Co-CNT/Ti_3_C_2_, Pt/C. (**e**) Chronoamperometric curves of Co-CNT/Ti_3_C_2_-60 and Pt/C. Adapted from [64], with permission from *Journal of Materials Chemistry A*, 2019. (**f**) Schematic for preparing of Fe-N_x_/N/Ti_3_C_2_. (**g**) Tafel plots of the catalysts. (**h**) n and peroxide species yield of the Fe-N_x_/N/Ti_3_C_2_ and commercial Pt/C. (**i**) i-t chronoamperometric response of Fe-N_x_/N/Ti_3_C_2_ and commercial Pt/C. (**j**) Comparison of E_onset_ and E_1/2_ of Fe-N_x_/N/Ti_3_C_2_with the reported ORR catalysts. Adapted from [65], with permission from *International Journal of Hydrogen Energy*, 2022.

Chen and colleagues reported a Co-CNT/Ti_3_C_2_ hybrid material synthesized via an in situ growth strategy, which was employed as an oxygen reduction reaction (ORR) electrocatalyst (Figure 9a). One-dimensional cobalt-embedded carbon nanotubes (1D Co-CNTs), derived from ZIF-67 precursors, were vertically aligned on two-dimensional Ti_3_C_2_ MXene nanosheets, forming a multidimensional heterostructure. Leveraging the synergistic effects between MXene nanosheets and Co-CNTs—including enhanced charge transfer and optimized intermediate adsorption—the Co-CNT/Ti_3_C_2_ hybrid demonstrated exceptional ORR performance with a half-wave potential of 0.82 V vs. RHE and a diffusion-limited current density of 5.55 mA cm^−2^, alongside enhanced stability (Figure 9b–e) [64]. Gu et al. synthesized Fe-N_x_/N-doped Ti_3_C_2_ (Fe-N_x_/N/Ti_3_C_2_) electrocatalysts by first growing MIL-Fe/Ti_3_C_2_ precursors in situ on MXene nanosheets, followed by sequential carbonization and nitridation (Figure 9f). The high electrical conductivity of MXene significantly accelerated ion/electron transfer kinetics, achieving a charge transfer resistance of 12 Ω—fourfold lower than that of non-MXene counterparts. Furthermore, strong electronic interactions within the multidimensional nanostructure enabled precise modulation of the catalytic center’s electronic configuration. The Fe-N_x_/N/Ti_3_C_2_ catalyst exhibits superior ORR catalytic activity, significantly lower H_2_O_2_ yield, and exceptional durability in both alkaline and acidic electrolytes compared to commercial Pt/C benchmarks (Figure 9g–j) [65].

### 4.2. Supercapacitors

Supercapacitors, characterized by their superior power density, rapid charging capability, and extended cycle lifespan, represent a highly promising class of energy storage devices [66,67]. Extensive optimization strategies have been implemented to address their primary limitations, including but not limited to modification of existing electrode materials, discovery of novel active materials, exploration of advanced electrolytes, optimization of device assembly protocols, and enhancement of operational voltage windows [68,69]. From a materials development perspective, while noble metal-based materials are considered ideal electrode candidates due to their exceptional specific capacitance, their prohibitive cost and scarcity severely restrict practical scalability [70]. MXene possesses a lamellar structure and high electrical conductivity, enabling rapid electrolyte penetration through its stacked layers. The abundant surface functional groups on MXene facilitate high-rate reversible redox reactions, and its hybridization with MOF-derived materials introduces synergistic pseudocapacitive contributions [71]. Furthermore, the multidimensional nanostructure of MXene/MOF-derived composites provides increased active site density and enhanced conductivity, which collectively optimize charge storage kinetics and structural stability during electrochemical cycling.

**Figure 10:**
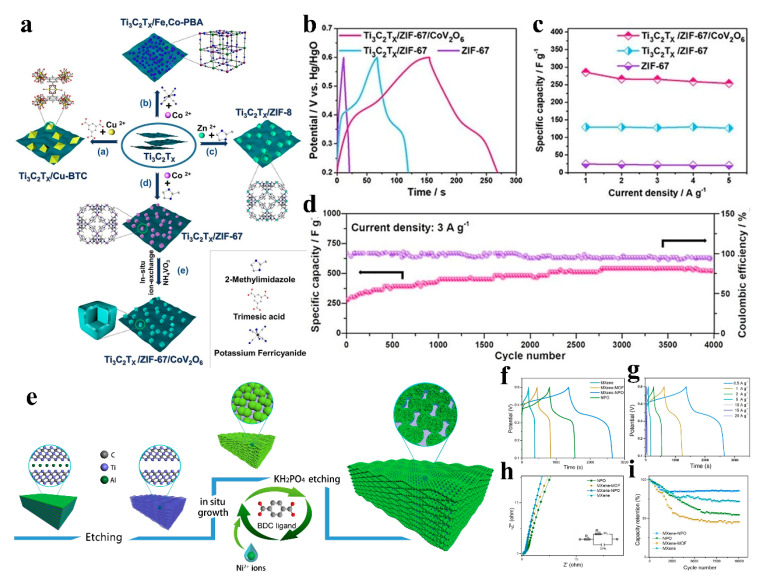
(**a**) Schematic for preparing of Ti_3_C_2_T_x_/ZIF-67/CoV_2_O_6_. (**b**) GCD curves of ZIF-67, Ti_3_C_2_T_X_/ZIF-67 and Ti_3_C_2_T_X_/ZIF-67/CoV_2_O6 at 1 A g^−1^. (**c**) Specific capacitance of ZIF-67, Ti_3_C_2_T_X_/ZIF-67 and Ti_3_C_2_T_X_/ZIF-67/CoV_2_O_6_ at current densities from 1to 5 A g^−1^. (**d**) Long-term cycling stability of Ti_3_C_2_T_X_/ZIF-67/CoV_2_O_6_ over 4000 GCD cycles at a current density of 3 A g^−1^. Adapted from [73], with permission from *Angewandte Chemie International Edition*, 2022. (**e**) Schematic for preparing of MXene/NPO. (**f**) GCD curves of the as-prepared electrode materials at 0.5 A g^−1^. (**g**) GCD curves of the MXene/NPO electrode at different current density. (**h**) Nyquist plots of (inset is the equivalent circuit). (**i**) The cycling tests of as-prepared electrodes at 10 A g^−1^. Adapted from [74], with permission from *Chemical Engineering Journal*, 2021.

S.R. Shingte’s team successfully synthesized Ti_3_C_2_ MXene/NFO composites with varying loading percentages of rhombohedral nickel ferrite nanoparticles (NFO NPs) for supercapacitor applications. The NFO NPs act as interlayer spacers between MXene sheets, enhancing layer separation and facilitating efficient charge transfer at the electrode–electrolyte interface. A synergistic interplay between MXene and NFO significantly improves the electrochemical performance. Notably, an optimal NFO NP loading percentage (8 wt%) was critical for maximizing the overall supercapacitive performance of the MXene/NFO thin-film electrodes. This multidimensional nanostructured composite material exhibits exceptional electrochemical performance. The MXene/NFO-8 electrode exhibited a C_SP_ of 660 F g^−1^ at the 1 A g^−1^. An all-solid-state supercapacitor device MXene/NFO-8//AC with PVA-HOH gel electrolyte demonstrated a high energy density of 17.36 Wh kg^−1^ at the power density of 718.48 W kg^−1^. The device retained ~70% of capacitance even after 5000 cycles [72]. Liu et al. constructed a three-dimensional porous Ti_3_C_2_T_x_/ZIF-67/CoV_2_O_6_ composite via an ion conversion and exchange strategy, which was employed as a high-performance supercapacitor electrode (Figure 10a). The Ti_3_C_2_T_x_/ZIF-67/CoV_2_O_6_ electrode demonstrated a specific capacitance of 285.5 F g^−1^ at 1 A g^−1^ and exceptional cycling stability, retaining 94.4% of its initial capacitance after 4000 cycles under the same current density (Figure 10b–d) [73]. Zhang’s team synthesized a multidimensional MXene-NPO composite through an in situ growth strategy to prepare MXene/Ni-MOF precursors, followed by phosphidation (Figure 10e). This multidimensional nanostructure facilitates rapid ion/electron migration at the NPO-MXene interfaces, leveraging synergistic interfacial interactions. The MXene/NPO electrode demonstrated a high specific capacity of 639 C g^−1^ at 1 A g^−1^ and exceptional long-term cycling stability. An asymmetric supercapacitor (ASC) was assembled using MXene/NPO//reduced graphene oxide (rGO) as the anode, PPD/rGO as the cathode, and KOH/PVA gel as the electrolyte. The MXene/NPO//rGO ASC achieved a high energy density of 72.6 Wh kg^−1^ at a power density of 932 W kg^−1^ (Figure 10f–i) [74].

### 4.3. Batteries

With the advancement of multifunctional electronic devices and the growing demand for clean energy storage, conventional battery systems are increasingly inadequate to meet escalating requirements, necessitating urgent development of portable rechargeable batteries with ultrahigh energy density [75,76,77]. MXene/MOF-derived composites, featuring multidimensional nanostructures, address these challenges by enhancing electrical conductivity and structural stability during charge/discharge processes. This architectural optimization facilitates rapid ion/electron transport kinetics, achieving a 2.8-fold improvement in rate capability and exceptional cycling stability [78].

#### 4.3.1. LIBs

**Figure 11:**
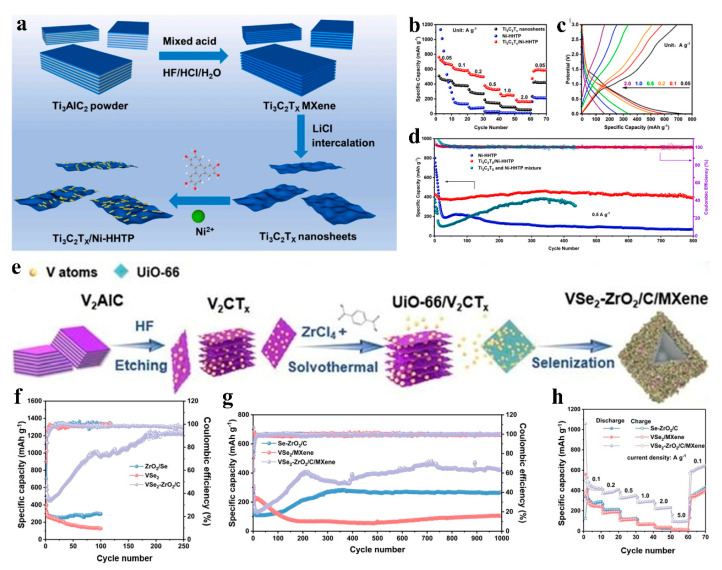
(**a**) Schematic for preparing of Ti_3_C_2_T_X_/Ni-HHTP. (**b**) Rate capability comparison among the cells with Ti_3_C_2_T_X_, Ni-HHTP, and Ti_3_C_2_T_X_/Ni-HHTP as anode materials. (**c**) Charge/discharge curves of the cell with Ti_3_C_2_T_X_/Ni-HHTP as anode material at different current densities. (**d**) Long-term cycling performance of the cell with three different kinds of anode materials at 0.5 A g^−1^. Adapted from [81], with permission from *Materials Today Communications*, 2024. (**e**) Schematic for preparing of Ti_3_C_2_T_X_/Ni-HHTP. (**f**) Cycling performances and corresponding Coulombic efficiencies of Se–ZrO_2_/C, VSe_2_/MXene, and VSe_2_–ZrO_2_/C/MXene at 100 mA g^−1^. (**g**) Long-term cyclabilities at 1.0 A g^−1^. (**h**) Rate performances of Se–ZrO_2_/C, VSe_2_/MXene and VSe_2_–ZrO_2_/C/MXene. Adapted from [82], with permission from *Journal of Materials Chemistry A*, 2023.

Lithium-ion batteries (LIBs), serving as well-established power sources for portable electronic devices such as smartphones, tablets, and laptops, play a pivotal role in modern society, particularly as critical enablers for energy sustainability due to their high energy density, scalable manufacturability, and compatibility with renewable energy integration strategies [79,80]. Wu et al. reported the synthesis of Ti_3_C_2_T_x_/Ni-HHTP composites for lithium-ion batteries via an in situ growth strategy to construct MXene/Ni-MOF hybrids, followed by controlled derivation (Figure 11a). The multidimensional heterostructured nanostructure formed between Ni-HHTP and Ti_3_C_2_T_x_ nanosheets facilitated rapid ion/electron transport and mitigated polarization during lithiation/delithiation, thereby enhancing electrochemical reaction kinetics. This conductive porous composite, featuring a high specific surface area and ordered mesoporous channels, demonstrated exceptional cycling stability and rate capability, delivering a reversible capacity of 390.2 mAh g^−1^ after 800 cycles at 0.5 A g^−1^ with 92.0% capacity retention (Figure 11b–d) [81]. Li et al. synthesized an octahedral VSe_2_-ZrO_2_/C/MXene composite derived from UiO-66/V_3_CT_x_ via a multi-step protocol (Figure 11e). Within this unique multidimensional nanostructure, the V_2_CT_x_-derived VSe_2_/MXene contributed a high reversible capacity, while ZrO_2_-assisted stabilization of VSe_2_ on octahedral carbon frameworks significantly enhanced electrochemical stability. Concurrently, the MOF-derived porous carbon matrix suppressed volume variation and improved Li^+^ transport kinetics. Consequently, the VSe_2_-ZrO_2_/C/MXene composite demonstrated an ultrahigh reversible capacity of 1238.5 mAh g^−1^ at 100 mA g^−1^ and retained 430 mAh g^−1^ after 1000 cycles at 1.0 A g^−1^, with a minimal capacity decay rate of 0.056% per cycle (Figure 11f–h) [82].

#### 4.3.2. SIBs

Over recent decades, sodium-ion batteries (SIBs) have emerged as one of the most promising alternatives to LIBs due to their low cost, natural abundance of sodium resources, and comparable high efficiency, garnering significant research attention [83]. However, single-component anode materials studied to date exhibit inherent limitations: Carbonaceous materials demonstrate exceptional long-term cycling stability, yet their low reversible capacity restricts practical deployment in high-energy-density SIBs. Conversely, metal oxides and alloy-based materials offer high specific capacities but suffer from rapid capacity decay and poor cycling longevity caused by substantial volume expansion during sodiation/desodiation. Consequently, the urgent development of ideal anode materials capable of balancing high capacity, structural resilience, and kinetic efficiency is imperative to advance next-generation energy storage systems [84,85,86]. The synergistic combination of MOF-derived materials with tunable pore architectures and MXene’s intrinsic [3] high conductivity enables MXene/MOF-derived composites featuring multidimensional nanostructures to effectively address the challenges encountered in sodium-ion battery (SIB) applications.

**Figure 12:**
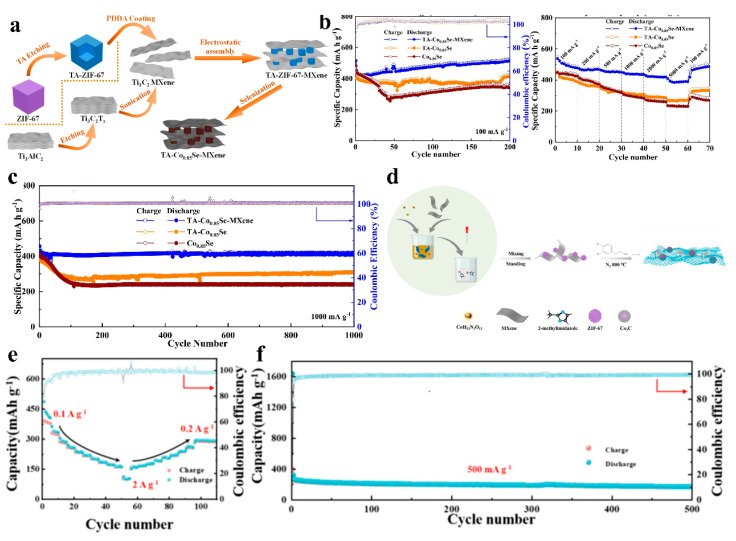
(**a**) Schematic for preparing of TA-Co_0.85_Se-MXene. (**b**) Cycling performance of TA-Co_0.85_Se-MXene, TA-Co_0.85_Se and Co_0.85_Se at 100 mA g^−1^ and rate properties of TA-Co_0.85_Se-MXene, TA-Co_0.85_Se and Co_0.85_Se at various current densities. (**c**) Long-term cycling performance of TA- Co_0.85_Se-MXene, TA- Co_0.85_Se and Co_0.85_Se at 1000 mA g^−1^. Adapted from [87], with permission from *Journal of Alloys and Compounds*, 2023. (**d**) Schematic for preparing of Co_3_C/MXene@C. (**e**) Rate performance of Co_3_C/MXene@C at various current densities from 100 to 2000 mA g^−1^. (**f**) Long-term cycling performance of Co_3_C/MXene@C at 500 mA g^−1^ for 500 cycles. Adapted from [88], with permission from *Colloids and Surfaces A: Physicochemical and Engineering Aspects*, 2023.

Shi’s team first synthesized ZIF-67 nanocubes, which were subsequently etched into hollow ZIF-67 nanocubes (denoted as TA-ZIF-67) using tannic acid (TA) as a structural modulator. The TA-ZIF-67 was then modified with PDDA and self-assembled with MXene nanosheets via electrostatic interactions. The resulting hybrid was subjected to carbonization and selenization to yield the final TA-Co_0.85_Se-MXene composite (Figure 12a). The TA-mediated deep etching of ZIF-67 created a hierarchical porous nanostructure, which not only alleviated volume variation during sodiation/desodiation but also enhanced reaction kinetics by shortening Na^+^ diffusion pathways. Strong interfacial interactions between MXene nanosheets and Co_0.85_Se nanocubes, achieved through the electrostatic self-assembly strategy, significantly accelerated Na^+^/electron transport. This meticulously engineered multidimensional TA-Co_0.85_Se-MXene demonstrated exceptional performance as a sodium-ion battery anode: an ultrahigh initial Coulombic efficiency (ICE) of 91%, outstanding rate capability (389 mAh g^−1^ at 5 A g^−1^), and remarkable long-term cyclability (418 mAh g^−1^ retained after 1000 cycles at 1 A g^−1^ with a minimal decay rate of 0.03% per cycle) (Figure 12b,c) [87]. Zhang et al. synthesized Co_3_C/MXene@C composites by dispersing MXene nanosheets with in situ-grown ZIF-67 (denoted as ZIF-67/MXene) in Tris buffer solution, followed by the addition of dopamine hydrochloride and overnight stirring. The mixture was subjected to high-temperature calcination to obtain the final Co_3_C/MXene@C composite (Figure 11d). During pyrolysis, ZIF-67 acted as a self-sacrificial template, transforming into highly conductive Co_3_C nanospheres anchored on MXene nanosheets, while dopamine hydrochloride-derived porous N-doped carbon enveloped the Co_3_C/MXene framework. This multidimensional nanostructure enhanced the composite’s overall electrical conductivity and mechanical robustness, enabling its application as a SIB anode. The material demonstrated a remarkable ICE of 98.43% and delivered an outstanding reversible specific capacity of 172 mAh g^−1^ with 89% capacity retention after 500 cycles (Figure 12d) [88].

#### 4.3.3. LSBs

**Figure 13:**
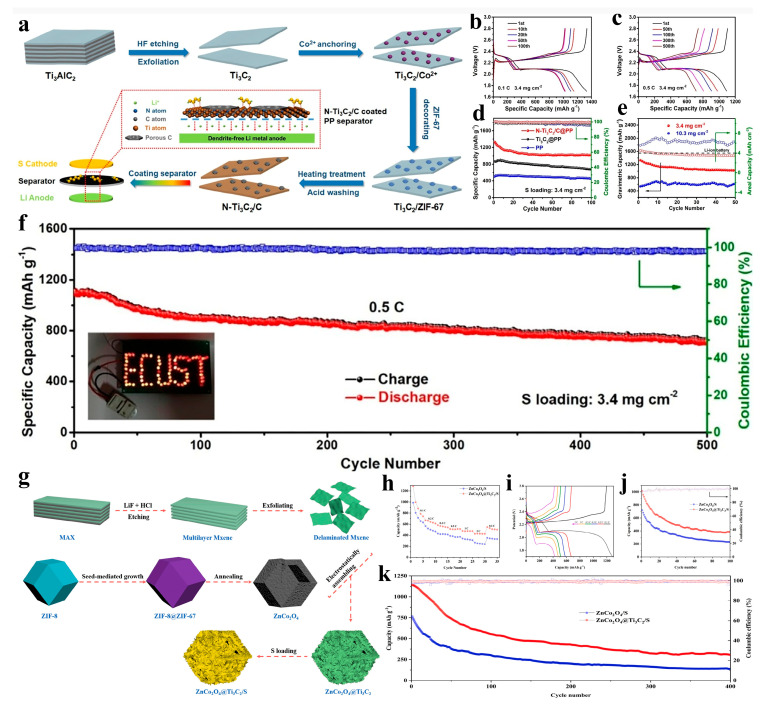
(**a**) Schematic for preparing of N- Ti_3_C_2_/C@The charge/discharge curves of the Li-S cell with N-Ti_3_C_2_/C@PP separator at (**b**) 0.1 C and (**c**) 0.5 C. (**d**) Cycling performances of Li-S cells with PP, Ti_3_C_2_@PP, and N-Ti_3_C_2_/C@PP separators at 0.1 C. (**e**) Cycling performances of Li-S cell with N-Ti_3_C_2_/C@PP separator under different sulfur-loading cathodes. (**f**) Long-term cycling performances of the Li-S cell with N-Ti_3_C_2_/C@PP separator at 0.5 C for 500 cycles. The inset in (**e**) shows the photograph of LED lamps with the pattern of “ECUST” powered by the Li-S cell with N-Ti_3_C_2_/C@PP separator. Adapted from [93], with permission from *Chemical Engineering Journal*, 2019. (**g**) Schematic for preparing of ZnCo_2_O_4_@Ti_3_C_2_/S. Cycling performances of ZnCo_2_O_4_@Ti_3_C_2_/S cathodes. (**h**) Rate performance of ZnCo_2_O_4_@Ti_3_C_2_/S and ZnCo_2_O_4_@Ti_3_C_2_ at current densities ranging from 0.1 C to 2.0 C, (**i**) The initial discharge/charge curves of ZnCo_2_O_4_@Ti_3_C_2_/S at various current rates. (**j**) Cycling performance of ZnCo_2_O_4_@Ti_3_C_2_/S cathodes at 0.2 C for 100 cycles. (**k**) The long-term cycling tests for ZnCo_2_O_4_@Ti_3_C_2_/S cathodes at 0.5 C. Adapted from [94], with permission from *Journal of Alloys and Compounds*, 2022.

Lithium-sulfur batteries (LSBs), owing to their high energy density and low material costs, represent a promising energy storage system for diverse emerging applications ranging from stationary grid storage to mobile electric vehicles [89]. However, the irreversible dissolution of higher-order polysulfide intermediates (Li_2_S_x_, 4 ≤ x ≤8) generated during lithium-ion intercalation/extraction processes directly compromises the cycling longevity of LSBs [90,91,92]. Multidimensional nanostructured MXene/MOF-derived composites address these challenges through two synergistic mechanisms: (i) robust physicochemical adsorption capabilities that effectively suppress polysulfide shuttling, and (ii) MXene’s expanded interlayer spacing and mechanical ductility, which accommodate substantial volume expansion of active sulfur species while maintaining structural integrity over extended cycling. Jiang et al. reported a novel porous carbon- and nitrogen-modified MXene nanosheet (N-Ti_3_C_2_/C) synthesized via in situ growth and pyrolysis of ZIF-67 on ultrathin MXene nanosheets, which was subsequently coated onto a commercial polypropylene (PP) separator to stabilize lithium-sulfur (Li-S) batteries (Figure 13a). ZIF-67 nanoparticles were uniformly decorated on Ti_3_C_2_ nanosheets, acting as spacers to prevent restacking while preserving their 2D geometry. Critically, the ZIF-67 modification did not fully cover the Ti_3_C_2_ surface, leaving exposed Lewis acid sites on Ti_3_C_2_ for chemical adsorption of polysulfides. Leveraging the synergistic effects of nitrogen-doping and porous carbon modification, the multidimensional N-Ti_3_C_2_/C-coated PP separator (N-Ti_3_C_2_/C@PP) not only effectively suppressed polysulfide shuttling but also inhibited lithium dendrite growth at the anode. The N-Ti_3_C_2_/C@PP-based Li-S battery exhibited exceptional electrochemical performance: a high reversible capacity of 1332 mAh g^−1^ at 0.1 C, long-term cycling stability (716 mAh g^−1^ retained after 500 cycles at 0.5 C with a sulfur loading of 3.4 mg cm^−2^), and a high areal capacity of 6.3 mAh cm^−2^ under an ultrahigh sulfur loading exceeding 3.4 mg cm^−2^ (Figure 13b–f) [93]. Wei et al. first prepared ZIF-8@ZIF-67 precursors and obtained ZnCo_2_O_4_ hollow polyhedron derivatives through high-temperature pyrolysis. These derivatives were then self-assembled with MXene nanosheets to form ZnCo_2_O_4_@MXene hybrid polyhedrons. Finally, the composite was thermally treated with sublimed sulfur to yield ZnCo_2_O_4_@Ti_3_C_2_/S (Figure 13g). This elaborately designed multidimensional nanostructure exhibits excellent electrochemical performance, attributed to the following: (i) the internal hollow structure not only provides additional sulfur storage sites (increasing sulfur loading and utilization) but also mitigates volume changes during charge/discharge processes; (ii) ZnCo_2_O_4_ offers sufficient polar active sites to suppress the dissolution and diffusion of LiPS, due to strong intermolecular interactions between polar ZnCo_2_O_4_ and LiPS; (iii) the conductive Ti_3_C_2_ enhances the electronic conductivity of the ZnCo_2_O_4_@Ti_3_C_2_ composite, thereby facilitating electron transfer. These advantages endow the ZnCo_2_O_4_@Ti_3_C_2_/S composite with superior reversible specific capacity and high-rate performance (Figure 13h–k) [94].

#### 4.3.4. ZABs

**Figure 14:**
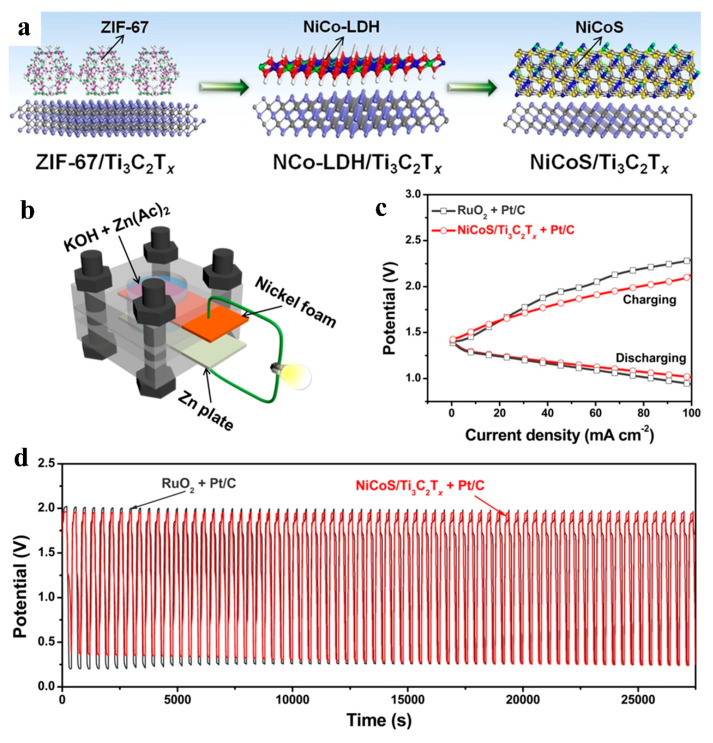
(**a**) Schematic for preparing of NiCoS/Ti_3_C_2_T_x_. (**b**) Scheme of a homemade rechargeable Zn−air battery. (**c**) Charging and discharging polarization curves of the rechargeable Zn−air batteries. (**d**) Cycling performance at the current density of 10 mA cm^−2^. Adapted from [100], with permission from *ACS Applied Materials & Interfaces*, 2018.

Zinc–air batteries (ZABs), with their ultrahigh theoretical specific energy density, intrinsic safety, and environmental benignity, offer immense potential for secure, clean, and decarbonized energy storage [95]. However, despite these merits—high energy density, low cost, and material abundance—the advancement of ZABs has been hindered by parasitic reactions at zinc anodes (e.g., hydrogen evolution and dendrite formation) and sluggish oxygen redox kinetics. Multidimensional nanostructured MXene/MOF-derived composites address these limitations through their high electrical conductivity, strong interfacial coupling, and large electrochemically active surface area. These attributes synergistically accelerate interfacial electron/ion transfer, reduce OER/ORR overpotentials, and suppress anode degradation mechanisms, thereby enabling stable, high-performance ZAB operation under practical conditions [96,97,98]. Qiao et al. synthesized CoNi-MOFs@MXene precursors via an in situ synthesis method. The precursors were thermally treated at 650 °C for 2 h under an argon atmosphere, followed by phosphorization using NaH_2_PO_2_∙H2O placed upstream at 300 °C for 2 h under argon flow, yielding the final H-CNP@M composite. This material features anchored heterogeneous multidimensional nanostructures, where CoNi-based nanoparticles (3–5 nm) are uniformly dispersed within a nitrogen/phosphorus-doped carbon matrix supported on a 3D MXene framework. The H-CNP@M composite demonstrates exceptional bifunctional catalytic activity, achieving E_1/2_ of 0.833 V vs. RHE for ORR and η_10_ of 294 mV for OER. When integrated into aqueous and all-solid-state ZABs, the H-CNP@M-based devices exhibit outstanding performance, delivering a peak power density of 166.5 mW/cm^2^ in aqueous electrolytes and stable cycling over 180 h with negligible voltage decay [99]. Zou et al. reported an MOF-derived/2D MXene hybrid synthesized via in situ growth of ZIF-67 on MXene nanosheets followed by derivation into hierarchically porous NiCoS hybrid metal sulfides (NiCoS/Ti_3_C_2_T_x_) (Figure 14a). The unique multidimensional nanostructure and intimate interfacial interactions between the two components not only ensured high electrical conductivity and enlarged surface area but also provided abundant active sites and exceptional oxygen evolution reaction (OER) activity. A homemade zinc–air battery was assembled using NiCoS/Ti_3_C_2_T_x_ coupled with Pt/C loaded on nickel foam as the cathode, a zinc plate as the anode, and 6 M KOH/0.2 M zinc acetate as the electrolyte. The device exhibited a low voltage gap of 0.7 V at 50 mA cm^−2^ with no decay after 8 h of constant-current charge/discharge cycling, demonstrating outstanding electrochemical stability (Figure 14b–d) [100].

Table 2 summarizes the electrochemical performance of MXene/MOF-derived composites with comparison to single components in Section 3 and Section 4.

## 5. Conclusions and Perspective

MOFs have garnered significant attention due to their abundant surface-active sites, high specific surface areas, and tunable pore architectures with adjustable porosity. While MOF-derived materials inherit these merits, their practical applications in energy storage systems remain constrained by inherent limitations, including poor structural stability and insufficient electrical conductivity. To address these challenges, researchers have pioneered innovative strategies centered on constructing multidimensional composite systems. MXene, with its exceptional electronic conductivity and rich surface functional groups, has emerged as a transformative component in such composites. Notably, MXene/MOF-derived hybrid systems engineered through molecular design strategies form multidimensional nanostructures, such as 0D nanoparticles confined within 1D nanotubes, 2D nanosheets or 3D interconnected frameworks that critically enhance structural stability and rate capability. Recent breakthroughs in materials nanoengineering have demonstrated that constructing multidimensional MXene/MOF-derived heterostructures can fundamentally overcome these limitations through synergistic architectural design. This review systematically examines the synthesis methodologies, performance metrics, and energy storage applications (electrocatalysts, supercapacitors, batteries) of multidimensional MXene/MOF-derived composites, with a focused analysis on how hierarchical nanostructuring dictates performance enhancements in ion diffusion kinetics, charge transfer efficiency, and mechanical resilience.

The design of synthesis pathways for MOF/MXene composites critically determines their performance and applicability [101]. Despite significant advancements in this field, several key challenges persist, such as the following: (i) achieving precise construction of MOF topological structures through metal node selection, ligand engineering, and microenvironment modulation to obtain ideal derived materials with tailored functionalities; (ii) developing environmentally benign MXene preparation protocols to enhance oxidation resistance and structural integrity, addressing the limitations of conventional HF-based etching methods [8]; (iii) engineering synergistic interfacial effects to establish “1 + 1 > 2” composite enhancement mechanisms, such as covalent bonding or electron redistribution at heterointerfaces [102,103]; (iv) utilizing in situ characterization techniques to dynamically resolve the evolution of multidimensional nanostructures during synthesis and operation [104]. Future research should focus on expanding MOF structural functionalization to transcend conventional framework limitations, developing novel multidimensional MOF architectures. Derivatives of such multidimensional MOFs, when hybridized with functional materials, can generate electrochemically active heterointerfaces that effectively enhance ion/electron transport kinetics through optimized interfacial charge transfer and lattice strain engineering. Deeper exploration of atomic-scale interface engineering strategies, such as covalent functional group modification or defect engineering, can further strengthen MXene/MOF-derived interactions, enabling the construction of robust multidimensional nanostructures. These architectures synergistically improve conductivity while enhancing stability against oxidative degradation. Concurrently, replacing traditional synthesis routes with sustainable fabrication methods is imperative. Innovations such as electrochemical exfoliation for MXene production, room-temperature MOF-MXene assembly via biomimetic mineralization, and microwave-assisted low-energy derivatization could reduce environmental impact by >60% while enabling scalable manufacturing [105]. Green synthesis strategies-leveraging aqueous solvents, renewable precursors, and energy-efficient processes-are critical for translating laboratory-scale breakthroughs into industrial applications.

Current research on multidimensional nanostructured MOF/MXene composites remains in its nascent stage, necessitating a “structure-determines-property” paradigm to guide rational material design and application. Despite persistent challenges in scalable fabrication and long-term stability, breakthroughs in energy storage applications hold promise through advanced in situ characterization techniques coupled with multiscale theoretical modeling. Continuous optimization of structural stability and functional tunability will critically underpin the development of MOF/MXene composites for next-generation energy storage systems.

## Figures and Tables

**Table 2:** Electrochemical performance summary of MXene/MOF-derived composites with comparison to single components.

Material	Performance	Comparison with Single Components	Testing Conditions	Application	Ref.
MXene@MOF/SA@H hydrogel framework	HER Tafel slope: 61.8 mV dec^−1^; OER Tafel slope: 84 mV dec^−1^; no degradation after 1000 cycles	—	Alkaline electrolyte, 25 °C	Electrocatalyst	[47]
3D@2D Co-Cu_3_P/NC@MXene	HER stability: No LSV curve decay after 1000 CV cycles	Cyclic stability outperformed non-hybridized Co-Cu_3_P/NC	0.5 M H_2_SO_4_ 5 mV s^−1^	Electrocatalyst	[48]
MXene@CoSe_2_@NC	203 mAh g^−1^ (20 A g^−1^, 5000 cycles)	Retention rate significantly higher than CoSe_2_@NC (Figure 8c)	0.01–3.0 V	SIBs	[50]
CoZnSe@N-MX	Ionic conductivity increased by 2.3× (vs. pristine MXene-based counterparts)	Direct comparison with pristine MXene-based materials	1 A g^−1^	SIBs	[53]
CoFe MLDH/Ti_3_C_2_/NF	OER overpotential optimization (DFT-confirmed interfacial charge redistribution)	Lower overpotential than pure CoFe LDH	1 M KOH 5 mV s^−1^	Electrocatalyst	[54]
MXene/NiCoZDH	Specific capacitance: 658 F g^−1^ (1 A g^−1^); 92% capacity retention after 5000 cycles	Higher capacitance than pristine MXene, ZIF-67, and non-composited NiCo-ZIF-67 (Figure 7d,e)	6 M KOH 0–0.5 V	Supercapacitor	[55]
Ti_3_C_2_ MXene/NFO-8	Specific capacitance: 660 F g^−1^ (1 A g^−1^); energy density: 17.36 Wh kg^−1^; 70% retention after 5000 cycles	Superior to pristine MXene and NFO-free electrodes	PVA-HOH gel electrolyte	Supercapacitor	[72]
Ti_3_C_2_T_x_/ZIF-67/CoV_2_O_6_	Specific capacitance: 285.5 F g^−1^ (1 A g^−1^); 94.4% retention after 4000 cycles	Higher capacitance than pristine ZIF-67 and CoV_2_O_6_-free composites (Figure 10b,c)	3 A g^−1^, KOH/PVA gel electrolyte	Supercapacitor	[73]
MXene/NPO	Specific capacity: 639 C g^−1^ (1 A g^−1^); energy density: 72.6 Wh kg^−1^	Higher capacity than non-composited NPO and MXene	KOH/PVA gel electrolyte, 1.6 V	Supercapacitor	[74]
Ti_3_C_2_T_x_/Ni-HHTP	Reversible capacity: 390.2 mAh g^−1^ (0.5 A g^−1^); 92% retention after 800 cycles	Outperformed pure Ni-HHTP and MXene (rate capability enhanced as shown in Figure 11b)	0.01–3.0 V	LIBs	[81]
VSe_2_-ZrO_2_/C/MXene	Reversible capacity: 1238.5 mAh g^−1^ (100 mA g^−1^); 430 mAh g^−1^ retained after 1000 cycles	Superior capacity and cyclability to non-composited VSe_2_ and ZrO_2_/C (Figure 11f–h)	1.0 A g^−1^, 0.01–3.0 V	LIBs	[82]
TA-Co_0.85_Se-MXene	Rate capability: 389 mAh g^−1^ (5 A g^−1^); 418 mAh g^−1^ retained after 1000 cycles (1 A g^−1^)	2×improved cyclability vs. pure Co_0.85_Se (Figure 12b)	0.01–3.0 V	SIBs	[87]
Co_3_C/MXene@C	Initial Coulombic efficiency: 98.43%; 89% capacity retention after 500 cycles	Initial Coulombic efficiency: 98.43%; 89% capacity retention after 500 cycles	500 mA g^−1^, 0.01–3.0 V	SIBs	[88]
N-Ti_3_C_2_/C@PP	Reversible capacity: 716 mAh g^−1^ (0.5 C); >80% retention after 500 cycles	Performance exceeds unmodified Ti_3_C_2_@PP and pure PP separators (Figure 13d–f)	Sulfur loading: 3.4 mg cm^−2^	LSBs	[93]
ZnCo_2_O_4_@Ti_3_C_2_/S	Initial discharge capacity: 1320 mAh g^−1^ (0.1 C); >85% retention after 100 cycles	Higher capacity retention than ZnCo_2_O_4_/S without MXene (Figure 13j,k)	0.2 C, 1.7–2.8 V	LSBs	[94]
NiCoS/Ti_3_C_2_T_x_	Voltage gap: 0.7 V (50 mA cm^−2^); no decay after 8 h of constant-current cycling	Lower overpotential than non-composited NiCoS	6 M KOH/ 0.2 M Zn(CH_3_COO)_2_ electrolyte	ZABs	[100]

## Data Availability

Data will be made available on request.
